# Glucometrics and Patient-Reported Outcomes in Individuals With Type 1 Diabetes Mellitus: Insights From the Correlation of Time in Range (CorrelaTIR) Study in Real-World Settings

**DOI:** 10.7759/cureus.79134

**Published:** 2025-02-17

**Authors:** Esther Artime, Natalia Hillman, Francisco J Tinahones, Antonio Pérez, Margarita Giménez, Natalia Duque, Miriam Rubio-De Santos, Silvia Díaz-Cerezo, Jennifer Redondo-Antón, Erik Spaepen, Francisco Pérez, Ignacio Conget

**Affiliations:** 1 Medicine, Eli Lilly and Company, Madrid, ESP; 2 Diabetes and Endocrinology, La Paz University Hospital, Madrid, ESP; 3 Diabetes and Endocrinology, Institute of Biomedical Research in Málaga (IBIMA), Hospital Virgen de la Victoria, Málaga, ESP; 4 Endocrinology and Nutrition, Hospital de la Santa Creu i Sant Pau, Barcelona, ESP; 5 Endocrinology and Nutrition, Hospital Clínic de Barcelona, Barcelona, ESP; 6 Statistics, HaaPACS GmbH, Schriesheim, DEU; 7 Diabetes and Endocrinology, Jaume I University, Madrid, ESP

**Keywords:** glucometrics, patient-reported outcomes, quality of life, time in range, type 1 diabetes mellitus

## Abstract

Background

This study aimed to measure the association between time in range (TIR) and other continuous glucose monitoring (CGM)-derived glucometrics, quality of life (QoL), healthcare resource use (HCRU), and costs in persons with type 1 diabetes mellitus (T1DM) in routine clinical practice in Spain.

Methods

This observational, cross-sectional, multicentre study evaluated persons with T1DM who received insulin via multiple daily injections. The study collected data on the participants (demographic and clinical), the use of the CGM devices, patient-reported outcomes (PROs) for general and diabetes-related QoL, treatment satisfaction, work productivity and activity impairment, HCRU, and costs. Data were analysed descriptively. The Spearman correlation coefficient was used to measure the association between glucometrics and PROs, HCRU and costs.

Results

Participants (N=114) had a mean age (standard deviation) of 44.53 (14.39) years, were 50.88% men, and 53.51% had glycated haemoglobin ≤7%. A higher TIR was significantly associated with better diabetes-related QoL but not with general QoL. HCRU and PRO scores for treatment satisfaction and work productivity and activity impairment showed no correlation with TIR. Higher TIR correlated with a lower number of emergency room visits.

Conclusion

Good glycaemic control (high TIR) is favourably associated with some aspects of diabetes-related QoL.

## Introduction

The introduction of continuous glucose monitoring (CGM) has radically changed the therapeutic management of persons with type 1 diabetes mellitus (T1DM) and how they achieve glycaemic control targets. Current CGM systems include both real-time CGM (rtCGM) and intermittently scanned CGM (isCGM), which can be combined with a regimen of multiple daily injections (MDIs) of insulin or a continuous subcutaneous insulin infusion (CSII) system [1,2]. Numerous studies have demonstrated significant clinical benefits of CGM in improving glycaemic control in persons with T1DM regardless of insulin delivery method [1]. The introduction of CGM devices can have an impact on healthcare costs by reducing the risk of hospitalisation for severe hypoglycaemia in both children and adults with T1DM [3,4].

CGM measures interstitial glucose concentrations and generates immediate information on glucose levels to the user [5,6], providing insights into the duration, frequency, and causes of fluctuations in blood glucose levels [2]. Glucometric parameters (GPs) derived from CGM measurements have become the gold standard to assess the achievement of glycaemic targets in persons with T1DM, beyond glycated haemoglobin (HbA1c) [7,8]. These parameters can quantify and represent time below (time below range, TBR), within (time in range, TIR), and above (time above range, TAR) the established glycaemic targets [7,9], and they can help facilitate discussions between patients and healthcare professionals at clinic visits [10]. The use of TIR (usually 70-180 mg/dL or 3.9-10.0 mmol/L) has become standard in clinical practice and has been endorsed by several national diabetes societies [9,11]. For most persons with diabetes, a TIR >70%, with a TBR <70 mg/dL of <4% and a TBR <54 mg/dL of <1% are recommended as targets, together with a TAR >180 mg/dL of <25% and TAR >250 mg/dL of <5% [9]. TIR is considered a predictor of diabetic microvascular complications and low TIR has been associated with increased risk of hospitalisation for hypoglycaemia or ketoacidosis [12,13].

The different GPs could have distinct and complex effects on the patient’s well-being and quality of life (QoL). For example, a pioneering study investigating how daily glycaemic control parameters are associated with mood in adults with T1DM found that TIR was an independent predictor of many positive and negative mood variables [14]. TIR is also associated with less fear of hypoglycaemia, a cause of lower overall health status and mental and physical health [15-17].

The evaluation of treatment satisfaction and patient well-being, or QoL, which are collectively called patient-reported outcomes (PROs), is becoming increasingly important in clinical practice and in the evaluation of healthcare services and technologies [18-22]. PROs place patients at the centre of their treatment, incorporating their perspectives on the management, helping in the detection of symptoms, preventing complications, and facilitating communication between patient and clinician. The use of PROs is recommended as we move towards a more personalised approach to diabetes care [23]. Although some studies suggest the positive impact of the introduction of CGM systems on QoL for persons with T1DM [20,24,25], there is still little evidence on the relationship between GP values and PROs [26]. Further, there is no evidence of the relationship between specific GPs and the use of healthcare resources or its associated costs.

The main objective of this study was to measure the relationship between TIR and QoL in persons with T1DM using CGM devices who receive insulin via MDI, in routine clinical practice, in Spain. The secondary objectives investigated were the association between TIR, TAR, and TBR with treatment satisfaction, productivity, and activity impairment; the demographic and clinical determinants; and the association between TIR, TAR, and TBR with healthcare resource use (HCRU) and associated costs.

This article was previously presented as a poster at the 64th Congress of the Spanish Society for Endocrinology and Nutrition (SEEN), held in Barcelona, Spain, on October 18-20, 2023.

## Materials and methods

The ‘Real-World Correlation of Time in Range With Patient-reported Outcomes and Healthcare Resource Use in Patients With Diabetes in Spain’ (CorrelaTIR) study was an observational, cross-sectional, multicentre study conducted in four hospitals with specialised diabetes units in Spain: Hospital Clinic (Barcelona), Hospital de la Santa Creu i Sant Pau (Barcelona), Hospital La Paz (Madrid), and Hospital Virgen de la Victoria (Málaga). These centres were selected as they routinely manage persons with T1DM using CGM systems, and CGM data are collected and incorporated into the electronic medical charts. The endocrinology units from these hospitals recruited participants consecutively from March to September 2022 as they attended routine visits. Participants were invited by the site medical team to complete a patient-electronic case report form (eCRF), which included health-related QoL, satisfaction with treatment, and productivity and activity impairment questionnaires. Additionally, the medical team extracted data from the electronic medical records and entered them in the physician-eCRF. Participation in the study did not change the routine treatment of the patients as determined by their treating physicians and all treatment decisions and type and timing of disease monitoring were at the discretion of the treating physician. No additional interventions or visits to the clinic were required for the purpose of the study. Neither the sponsor nor the Contract Research Organisation in charge of the study had access to any patient identification (ID) data; only the site personnel had access to this information. Data collected from patients was kept de-identified via an ID, keeping the link between physician-eCRF and patient-eCRF. Participants in the study provided written informed consent and the study was approved by the Ethical Review Board of the Hospital Clinic of Barcelona (Reg. HCB/2021/0997).

Study population

The study included adults aged ≥18 years and with a diagnosis of T1DM who were on treatment with MDI of insulin and had used a CGM device (rtCGM or isCGM) for at least six months before the start of the study at the time of inclusion. In addition, the participants had to have available CGM data over the predefined period (14 days before inclusion), with ≥70% use of CGM, and had to provide signed informed consent. Persons with T1DM who were receiving insulin treatment via CSII, or could not complete the tasks of the study, were excluded. Also excluded were those needing translation of the study questionnaires, or those currently participating in a clinical trial.

Data collection and variables

**Figure 1:**
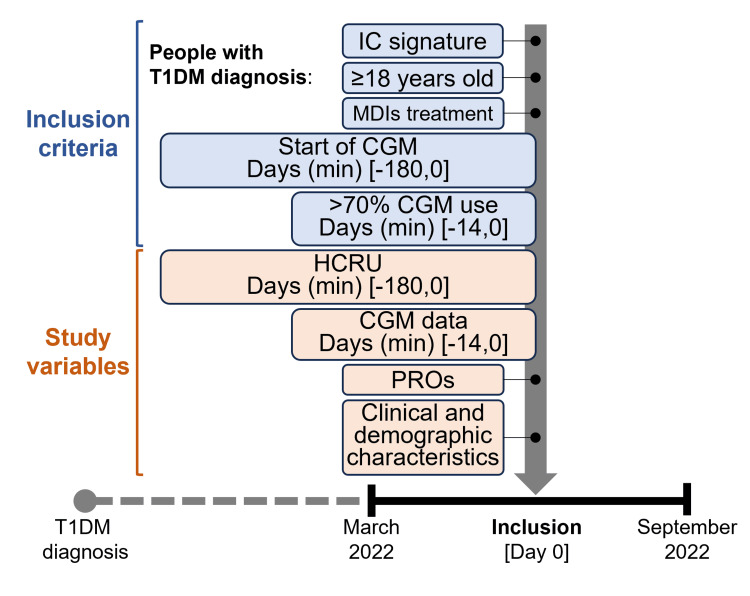
Study design The time period analysed to verify compliance with study selection criteria and to collect the different study variables is shown between brackets. CGM: continuous glucose monitoring; HCRU: health care resource use; IC: informed consent; MDIs: multiple daily injections; PROs: patient-reported outcomes; T1DM: type 1 diabetes mellitus

Data were obtained during a routine visit, in which study participants were invited to complete PROs and the medical team extracted data from electronic medical records: sociodemographic, clinical (body mass index (BMI), date of T1DM diagnosis, HbA1c (most recent value, within one year of inclusion), comorbidities, awareness of hypoglycaemia), treatment-related data (insulin type and dose, antidiabetic treatments, and concomitant treatments), HCRU in the previous six months, data from first time of CGM use, and GPs from the last 14 days before recruitment (Figure 1).

GPs related to diabetes control comprised the glucose management indicator (GMI; a parameter estimating HbA1c levels based on the average CGM readings for 14 or more days), TIR, TAR, TBR, mean glucose (average glucose levels in a given period), and glycaemic variability (percentage of coefficient of variation (%CV)) [9,11]. Data related to the use of the CGM device included the start date of CGM prescription, the main reason for initiating CGM, and the type of CGM device (rtCGM or isCGM and support app). The number of specialised care consultations, emergency room visits, and hospital stays in the previous six months were collected, and the associated costs were obtained by multiplying the number of times each resource had been used by their unit cost. Unitary costs of HCRU were obtained from eSalud, a health costs database [27]. The cost of treatment was not included.

The following general and diabetes-specific PRO instruments were self-administered to the participants (Table 1): the European Quality of Life-5 Dimensions-5 Levels questionnaire (EQ-5D-5L) [28]; the European Quality of Life-Visual Analogue Scale (EQ-VAS) [29]; the Diabetes Quality of Life Measure (DQOL) [30]; the Diabetes Treatment Satisfaction Questionnaire (DTSQ) [31]; and the Work Productivity and Activity Impairment (WPAI) questionnaire [32]. The EQ-5D-5L, EQ-VAS, and the WPAI questionnaire are generic PROs, while the DQOL and DTSQ are diabetes-specific. Spanish-validated versions of these instruments were used in all cases. For the EQ-5D-5L, EQ-VAS, and DTSQ, a higher score indicates better QoL. For the DQOL and WPAI, a higher score indicates worse QoL and lower productivity/greater impairment, respectively. The PRO questionnaires had to be completed by the participants within 15 days of inclusion (when CGM data were downloaded).

**Table 1:** PROs used in this study PROs: patient-reported outcomes; HRQoL: health-related quality of life

Name, reference	Description
European Quality of Life-5 Dimensions-5 Levels questionnaire (EQ-5D-5L) [28]	EuroQoL-5D (EQ-5D-5L) is a standardised generic instrument used to measure overall health status. It provides both a descriptive profile and a single index value for health status. This questionnaire evaluates five dimensions: mobility; self-care; usual activities; pain/discomfort; and anxiety/depression. Each dimension has five levels of severity: no problems; slight problems; moderate problems; severe problems; and extreme problems. The patient is asked to indicate their health state by ticking the box next to the most appropriate statement in each of the five dimensions. This decision results in a one-digit number that expresses the level selected for that dimension. The digits for the five dimensions can be combined into a five-digit number that describes the patient's health state.
European Quality of Life-Visual Analogue Scale (EQ-VAS) [29]	A visual analogue scale of EQ-5D-5L also assesses the patient's overall health state.
Diabetes Quality of Life Measure (DQOL) [30]	The DQOL is the most widely used specific questionnaire for measuring HRQoL in diabetes. The questionnaire consists of 46 questions distributed in four dimensions: ‘Satisfaction’ (15 questions); ‘Impact’ (20 questions); ‘Social/vocational concern’ (seven questions); and ‘Diabetes-related concern’ (four questions). Responses are quantified using a Likert scale of five ordinal responses. Each subject's total score on the scale is the sum of the scores given to each question in the questionnaire.
Diabetes Treatment Satisfaction Questionnaire (DTSQ) [31]	DTSQ is a questionnaire designed to measure satisfaction (status version) and change in satisfaction (change version) with diabetes treatment in patients with type 1 and type 2 diabetes mellitus. The status version is used to assess satisfaction with diabetes treatment and the perceived frequency of hyperglycaemia and hypoglycaemia at a given point in time. It consists of eight items, each of which is scored on a seven-point scale. The satisfaction score is the sum of the six sections of the questionnaire. Each section is scored from 6 to 0, so it can range from ‘very satisfied’ (36 points) to ‘very dissatisfied’ (0 points). The remaining two items measure the perceived frequency of hyperglycaemia and hypoglycaemia and are scored separately. They are scored from 0 (never perceived) to 6 (almost all the time).
Work Productivity and Activity Impairment (WPAI) questionnaire [32]	The WPAI questionnaire measures work time missed and work and activity impairment due to general health problems (WPAI:GH) during the past seven days. The WPAI:GH consists of six questions that elicit: employment status; hours missed due to health problems; hours missed due to other reasons; hours actually worked; and two questions that measure the degree health problems affected productivity while working (presenteeism) and regular daily activities, on a scale from 0 to 10. Scores for absenteeism, presenteeism, overall work productivity loss (combined absenteeism plus presenteeism), and impairment in regular daily (non-work) activities are derived for the interval of the past seven days; scores are expressed as a percentage of impairment/productivity loss, with higher scores indicating greater impairment.

Statistical analysis

The sample size was estimated assuming a conservative approach of a relatively low expected correlation (correlation coefficient = 0.35) between TIR and QoL. A minimum sample size of between 65 (80% power) and 105 (95% power) persons with T1DM was required. Considering a response rate of around 60%, 109 and 175 persons with T1DM were necessary to reach the required sample size, resulting in a power of 80% and 95%, respectively.

A descriptive analysis of the study variables, based on valid data, was performed (there was no imputing of missing data). Statistical analyses used Stata software Version 14 (StataCorp LLC, College Station, TX, USA). Qualitative variables were described by relative and absolute frequencies, and quantitative variables were described with centrality and dispersion measures (mean and standard deviation (SD)). A simple linear regression model, with Spearman correlation coefficient factor and resulting p-value, was used to analyse the relationship of GPs with the different PROs evaluated and HCRU and the associated total cost, according to different thresholds of GPs (TIR, TAR, TBR) and HbA1c (≤7% vs >7%).

## Results

Of 164 adults recruited for the study who met the selection criteria, 114 (69.5%) completed all the questionnaires and were included in the analysis. The characteristics of participants are provided in Table 2. The mean (SD) age of the participants was 44.5 (14.4) years, and 85% of them were under the age of 60 years; 58 (50.9%) were male. The mean (SD) time since T1DM diagnosis was 21.7 (13.8) years.

**Table 2:** Demographic and clinical characteristics of the study population ^1^ Calculated using cost data from the Oblikue database (http://oblikue.com/bddcostes/) updated to 2022 [27]. Cost derived from the use of healthcare resources in the previous six months, calculated by multiplying the number of times each resource has been used by its unit cost (excluding the cost of treatment). BMI: body mass index; HbA1c: glycated haemoglobin; HCRU: healthcare resource use; NA: not applicable; SD: standard deviation; T1DM: type 1 diabetes mellitus; UI: units of insulin

Variable	N=114
Age (years), mean (SD)	44.5 (14.4)
Sex, n (%)	
Male	58 (50.9)
Female	56 (49.1)
Years from T1DM diagnosis, mean (SD)	21.7 (13.8)
Mean glucose (mg/dL), mean (SD)	157.2 (27.6)
HbA1c (%), mean (SD)	7.1 (0.9)
HbA1c (mmol/mol), mean	54.1
HbA1c <7% (<53 mmol/mol), n (%)	61 (53.5)
BMI (kg/m^2^), mean (SD)	25.9 (5.2)
Number of comorbidities (n), mean (SD)	1.6 (1.5)
Participants with at least one comorbidity, n (%)	82 (71.9)
1	31 (27.2)
2	23 (20.2)
≥3	28 (24.6)
Main comorbidities, n (%)	
Dyslipidaemia	51 (44.7)
High blood pressure	25 (21.9)
Diabetic retinopathy	19 (16.7)
Obesity	15 (13.2)
Total insulin daily dose (UI), mean (SD)	46.3 (22.3)
Basal (n=113)	23.3 (12.2)
Rapid (n=113)	23.2 (14.1)
Pre-mixed (n=1)	27 (NA)
Use of concomitant non-glucose lowering medications, n (%)	79 (69.3)
Statins	47 (41.2)
Anti-hypertensives	26 (22.8)
Antibiotics	20 (17.5)
Hormonal therapy	20 (17.5)
HCRU and associated costs in the previous six months	
Number of visits to specialised care, mean (SD)	2.1 (1.0)
Number of calls to specialised care, mean (SD)	0.13 (0.39)
Number of visits to the emergency room, mean (SD)	0.04 (0.30)
Total cost, Euros^1^, mean (SD)^1^	280.14 (152.04)

CGM use and diabetes control

**Figure 2:**
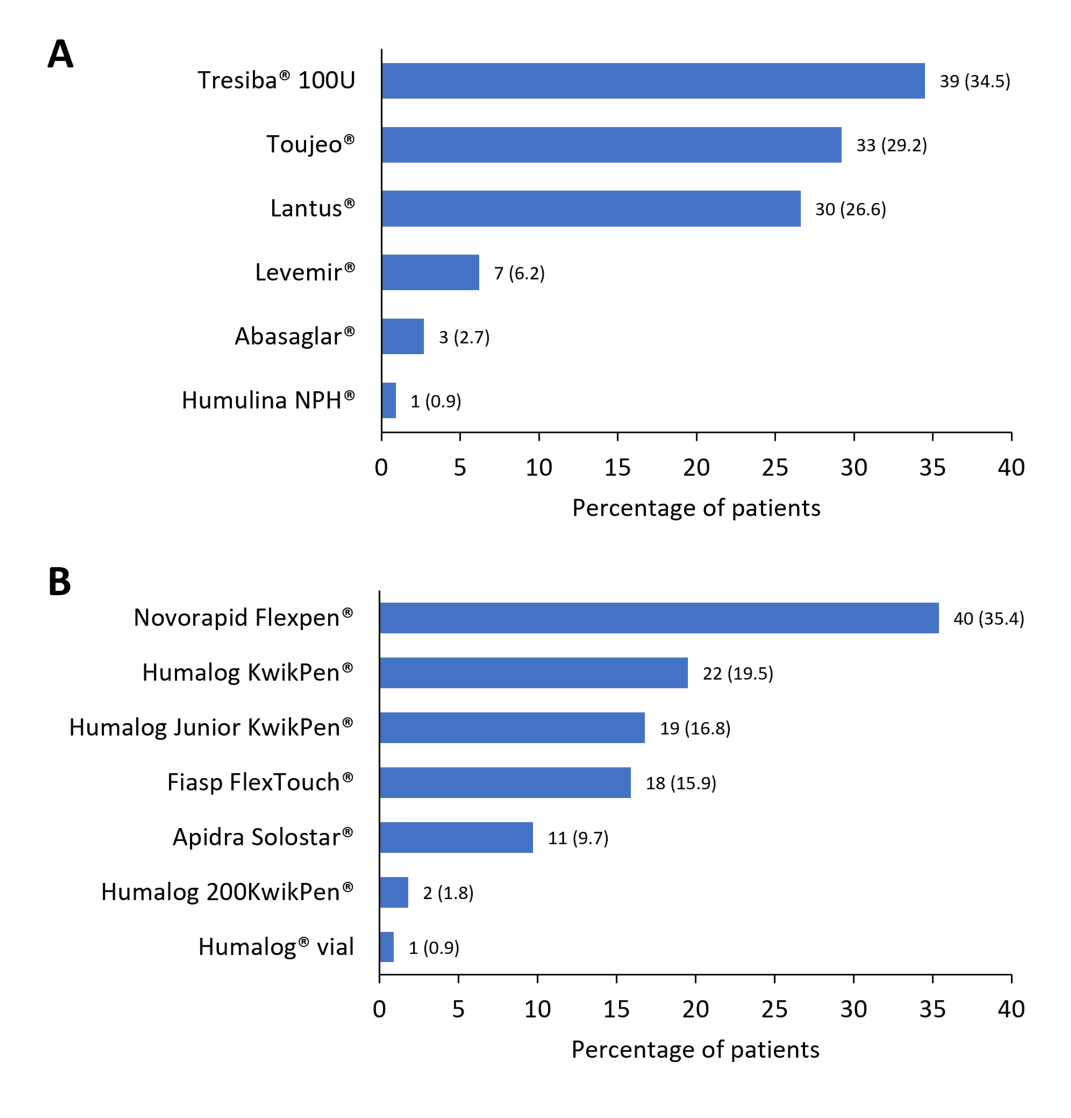
Frequency of use of basal (A) and rapid (B) insulin treatments Basal insulin and rapid insulin, N=113; pre-mix insulin, N=1; total insulin daily dose, N=114

**Figure 3:**
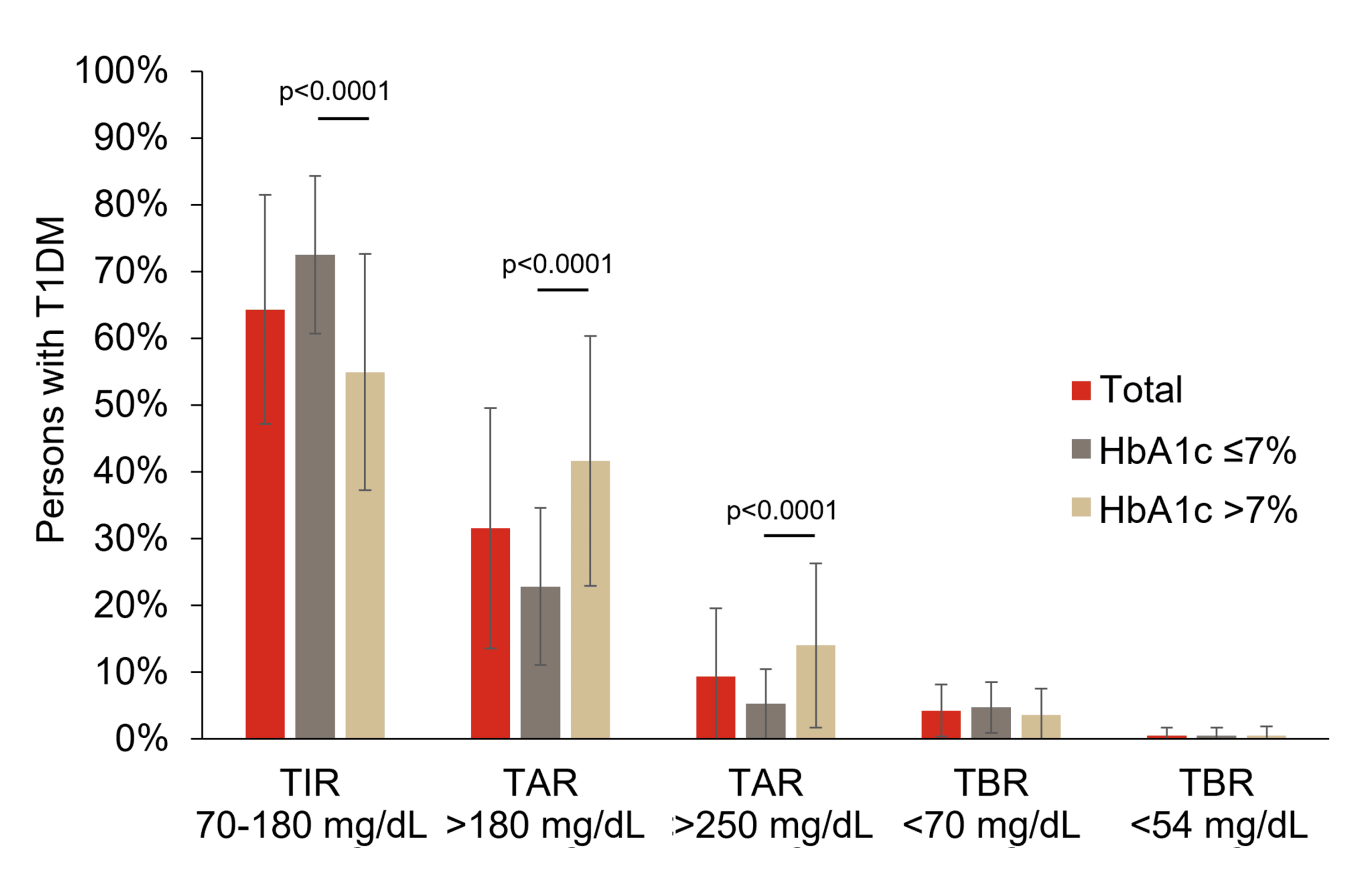
Glycometric parameters (last 14 days) The t-test was used to analyse the differences between subgroups (HbA1c ≤7% and HbA1c >7%). HbA1c: glycated haemoglobin; T1DM: type 1 diabetes mellitus; TAR: time above range; TBR: time below range; TIR: time in range

The mean (SD) time using the CGM devices was 1.8 (1.9) years, with Flash devices being the most commonly used (98.3%, n=112) (Table 3). The primary reason for initiating CGM was the diagnosis of T1DM (59.7%, n=68), followed by recurrent hypoglycaemia (21.9%, n=25) and poor metabolic control (18.4%, n=21) (Table 3). Mean (SD) TIR (70-180 mg/dL) was 64.3 (17.2%), and the TIR was higher than 70% in 38.6% (n=44) of participants. The population presented a mean HbA1c of 7.1% (54.1 mmol/mol), and over half of the participants (53.5%) were classified as HbA1c controlled (HbA1c ≤7%) (Table 2). The main findings related to T1DM control are shown in Table 3. The frequency of use of the different basal and rapid insulins is presented in Figure 2. Significantly higher TIR and lower TAR values (p<0.0001) were observed in the HbA1c ≤7% subgroup (Figure 3).

**Table 3:** T1DM-related clinical parameters and control variables of the study population CGM: continuous glucose monitoring; CV: coefficient of variation; GMI: glucose management indicator; T1DM: type 1 diabetes mellitus; TAR_1_: time above range (glucose >180 mg/dL); TAR_2_: time above range (glucose >250 mg/dL); TBR_1_: time below range (glucose <70 mg/dL); TBR_2_: time below range (glucose <54 mg/dL); TIR: time in range (glucose >70 and <180 mg/dL); SD: standard deviation

Variable	N=114
Percent of TIR, mean (SD)	64.3 (17.2)
Percent of TAR_1_, mean (SD)	31.5 (18.0)
Percent of TAR_2_, mean (SD)	9.3 (10.2)
Percent of TBR_1_, mean (SD)	4.2 (3.9)
Percent of TBR_2_, mean (SD)	0.5 (1.2)
Serum glucose (mg/dL), mean (SD)	157.2 (27.6)
Glycaemic variability, CV (%), mean (SD)	35.7 (6.2)
GMI (%), mean (SD)	7.2 (1.4)
TIR >70%, n (%)	44 (38.6)
Years with CGM, mean (SD)	1.8 (1.9)
Main reason for initiating CGM, n (%)	
T1DM (in general)	68 (59.7)
Recurrent hypoglycaemia	25 (21.9)
Poor metabolic control	21 (18.4)
Device used, n (%)	
Flash device	112 (98.2)
Real-time device	2 (1.8)

PROs

The mean scores for the questionnaires completed by the participants are presented in Table 4. Scores for general (EQ-5D-5L (-1,1), >0.8) and diabetes-specific QoL (DQOL (43-215), <90) and treatment satisfaction (DTSQ (0-36), >28) were high. Neither work productivity nor the ability to carry out regular daily activities (WPAI (0-1) <0.2) were reported to be highly impaired.

**Table 4:** Overall results of quantitative PROs variables (N=114) ^1^ A higher score indicates better QoL. ^2^ EQ-VAS data were missing from two participants. ^3^ A higher score indicates worse QoL. ^4^ A higher score indicates worse productivity and impairment. DQOL: Diabetes Quality of Life Measure; DTSQ: Diabetes Treatment Satisfaction Questionnaire; EQ-5D-5L: European Quality of Life-5 Dimensions-5 Levels questionnaire; EQ-VAS: European Quality of Life-Visual Analogue Scale; PROs: patient-reported outcomes; QoL: quality of life; SD: standard deviation; WPAI: Work Productivity and Activity Impairment

Questionnaire (range)	Mean (SD)
EQ-5D-5L index score (-1 to 1)^1^	0.88 (0.18)
EQ-VAS (0-100)^1,2^	78.06 (16.06)
DTSQ satisfaction (0-36)^1^	28.18 (5.20)
DTSQ hyperglycaemia (0-6)^1^	2.72 (1.52)
DTSQ hypoglycaemia (0-6)^1^	2.05 (1.34)
DQOL (43-215)^3^	87.90 (19.27)
DQOL satisfaction (15-75)^3^	32.75 (7.92)
DQOL impact (17-85)^3^	34.07 (8.59)
DQOL social/vocational concern (7-35)^3^	11.98 (4.10)
DQOL diabetes-related concern (4-20)^3^	9.11 (2.40)
WPAI absenteeism (0-1)^4^	0.04 (0.16)
WPAI presenteeism (0-1)^4^	0.07 (0.12)
WPAI overall work impairment (0-1)^4^	0.08 (0.13)
WPAI regular daily activities (0-1)^4^	0.17 (0.26)

HCRU and associated costs

Participants reported a mean (SD) of 2.1 (1.0) and 0.13 (0.39) visits to specialised care and calls with the endocrine unit in the six months prior to the study, respectively (Table 2). Most of the participants had registered at least one (25.4%, n=29) or two (48.3%, n=55) visits. The mean (SD) HCRU-associated cost per participant was estimated at 280.14 Euros (152.04).

Association between TIR, TAR, and TBR with PROs and HRCU

A weak but significant negative Spearman correlation coefficient (r_s_=-0.2004, p=0.0325) between TIR and DQOL scores was found, indicating that higher TIR values are associated with lower scores for DQOL, suggesting a better diabetes-related QoL. This was also observed for two of its subscales: satisfaction and diabetes-related concern (r_s_=-0.2105, p=0.0246; and r_s_=-0.2321, p=0.0130, respectively). However, a r_s_=0.0754 (p=0.4252) was obtained between TIR and EQ-5D-5L scores (index score), without statistical significance (Table 5 according to GP thresholds; Table 6 according to HbA1c thresholds). No significant association was found between TIR and treatment satisfaction, measured by DTSQ, or with work productivity and activity impairment, as measured by the WPAI questionnaire.

**Table 5:** Correlation between TIR, TAR and TBR with QoL and HCRU All values represented are the Spearman correlation coefficient and the p-value (in parentheses). Statistically significant associations (p <0.05) are highlighted in bold. DQOL: Diabetes Quality of Life Measure; DTSQ: Diabetes Treatment Satisfaction Questionnaire; EQ-5D-5L: European Quality of Life-5 Dimensions-5 Levels questionnaire; EQ-VAS: European Quality of Life-Visual Analogue Scale; ER: emergency room; HCRU: healthcare resource use; QoL: quality of life; SD; standard deviation; TAR: time above range; TIR: time in range; TBR: time below range; WPAI: Work Productivity and Activity Impairment

Variable	TIR (>70, <180 mg/dL)	TAR_1_ (>180 mg/dL)	TAR_2_ (>250 mg/dL)	TBR_1_ (<70 mg/dL)	TBR_2_ (<54 mg/dL)
EQ-5D-5L	0.0754 (0.4252)	-0.0635 (0.5018)	-0.0142 (0.8811)	0.0600 (0.5259)	-0.0919 (0.3307)
EQ-VAS	0.1172 (0.2186)	-0.1208 (0.2044)	-0.0752 (0.4309)	0.0538 (0.5735)	-0.0181 (0.8502)
DTSQ satisfaction	0.0949 (0.3151)	-0.0657 (0.4872)	-0.0753 (0.4258)	-0.0410 (0.6647)	-0.0103 (0.9133)
DQOL total score	-0.2004 (0.0325)	0.1859 (0.0477)	0.1715 (0.0681)	-0.0118 (0.9007)	0.0193 (0.8381)
DQOL satisfaction	-0.2105 (0.0246)	0.1924 (0.0403)	0.1445 (0.1252)	-0.0255 (0.7875)	0.0209 (0.8252)
DQOL impact	-0.1174 (0.2134)	0.1314 (0.1634)	0.1373 (0.1452)	-0.0699 (0.4601)	-0.0345 (0.7156)
DQOL social/vocational concern	-0.1016 (0.2819)	0.0726 (0.4424)	0.1135 (0.2292)	0.0597 (0.5278)	0.0458 (0.6288)
DQOL diabetes-related concern	-0.2321 (0.0130)	0.2143 (0.0220)	0.1502 (0.1106)	-0.0240 (0.7996)	0.0298 (0.7532)
WPAI absenteeism	-0.2068 (0.0751)	0.2017 (0.0827)	0.0797 (0.4969)	0.0433 (0.7121)	-0.0263 (0.8228)
WPAI presenteeism	-0.0567 (0.6335)	0.0787 (0.5082)	0.0459 (0.6998)	-0.0221 (0.8531)	0.0863 (0.4681)
WPAI overall work impairment	-0.0805 (0.4983)	0.0916 (0.4407)	0.0128 (0.9147)	0.0033 (0.9776)	0.0729 (0.5398)
WPAI regular daily activities	0.1204 (0.2019)	-0.0846 (0.3706)	-0.1182 (0.210 4)	-0.0951 (0.3141)	-0.0184 (0.8459)
Number of visits	-0.0304 (0.7484)	0.0468 (0.6212)	0.0832 (0.3786)	-0.1020 (0.2804)	0.0296 (0.7549)
Number of calls	0.0340 (0.7192)	-0.0433 (0.6477)	-0.0026 (0.9778)	0.0551 (0.5603)	-0.0318 (0.7370)
Number of ER visits	-0.2104 (0.0247)	0.1751 (0.0624)	0.2097 (0.0251)	0.0145 (0.8780)	0.1022 (0.2793)
HCRU-associated cost	-0.0386 (0.6837)	0.0421 (0.6567)	0.0937 (0.3212)	-0.0597 (0.5281)	0.0729 (0.4406)

**Table 6:** Correlation between TIR and QoL and HCRU for the total population and for the HbA1c ≤7% and >7% subgroups All values represented are the Spearman correlation coefficient and p-value (in parentheses). Statistically significant associations (p <0.05) are highlighted in bold. * Normality was found; hence the Pearson coefficient was calculated. DQOL: Diabetes Quality of Life Measure; DTSQ: Diabetes Treatment Satisfaction Questionnaire; EQ-5D-5L: European Quality of Life-5 Dimensions-5 Levels questionnaire; EQ-VAS: European Quality of Life-Visual Analogue Scale; ER: emergency room; HCRU: healthcare resource use; QoL: quality of life; SD: standard deviation; TAR: time above range; TIR: time in range; TBR: time below range; WPAI: Work Productivity and Activity Impairment

Variable	Total (n=114)	HbA1c ≤7% (n=61)	HbA1c >7% (n=53)
EQ-5D-5L	0.0754 (0.4252)	0.0830 (0.5247)	0.0638 (0.6500)
EQ-VAS	0.1172 (0.2186)	0.0837 (0.5249)	0.2301* (0.1008)
DTSQ satisfaction	0.0949 (0.3151)	0.0061 (0.9625)	0.0443* (0.7528)
DQOL total score	-0.2004 (0.0325)	-0.1209 (0.3533)	-0.2528 (0.0678)
DQOL satisfaction	-0.2105 (0.0246)	-0.0625* (0.6321)	-0.2543* (0.0661)
DQOL impact	-0.1174 (0.2134)	-0.0807 (0.5363)	-0.1805 (0.1959)
DQOL social/vocational concern	-0.1016 (0.2819)	-0.1399 (0.2823)	-0.2058 (0.1393)
DQOL diabetes-related concern	-0.2321 (0.0130)	-0.1130* (0.3860)	-0.3146* (0.0218)
WPAI absenteeism	-0.2068 (0.0751)	-0.2817 (0.0707)	0.0583 (0.7471)
WPAI presenteeism	-0.0567 (0.6335)	-0.1203 (0.4536)	0.0097 (0.9581)
WPAI overall work impairment	-0.0805 (0.4983)	-0.1662 (0.2990)	0.0495 (0.7881)
WPAI regular daily activities	0.1204 (0.2019)	0.0209 (0.8731)	0.0993 (0.4794)
Number of visits	-0.0304 (0.7484)	-	-
Number of calls	0.0340 (0.7192)	-	-
Number of ER visits	-0.2104 (0.0247)	-	-
HCRU-associated cost	-0.0386 (0.6837)	-0.1439 (0.2686)	-0.0179 (0.8986)

With respect to TAR and TBR, only TAR1 showed a statistically significant positive correlation with the DQOL total score (r_s_=0.1859, p=0.0477), as well as with two of its subscales: satisfaction and diabetes-related concern (r_s_=0.1924, p=0.0403; and r_s_=0.2143, p=0.0220, respectively). As the percentage of TAR1 decreases, the DQOL score is lower, indicating a better QoL. No associations were found between TAR or TBR with the DTSQ or the WPAI questionnaire.

Regarding correlations with HCRU, a statistically significant association with the number of emergency room visits was observed for TIR (r_s_=-0.2104, p=0.0247) and TAR2 (r_s_=0.2097, p=0.0251). However, it should be noted that only two participants required emergency visits during the six months prior to the study, with a total of four emergency room visits (one participant attended the emergency medical service once and the other participant required three different visits). No association was observed between TIR, TAR, or TBR with the number of visits, the number of calls, or the total costs. No association was found between GMI and the coefficient of variation with PROs.

## Discussion

This study evaluated the correlation between TIR, TAR, and TBR with QoL and HCRU in a population of persons with T1DM using CGM devices and receiving insulin via MDI. The results confirmed previous research and showed a weak but significant negative correlation between TIR and QoL as measured by the diabetes-specific questionnaire DQOL. Hence, as the percentage of TIR increased (better overall glycaemic control), the DQOL total score decreased (higher QoL). Likewise, the scores for DQOL subscales of diabetes-related concern and satisfaction were found to be significantly correlated with TIR. However, correlations between TIR and EQ-5D-5L (index score) and EQ-VAS were not significant. Other PROs, such as treatment satisfaction (DTSQ) and activity impairment, and HCRU, showed no correlation with TIR.

A possible explanation for the weak correlations found in our study could be that the population with T1DM treated with MDI and attended by specialised teams in the different participating centres had mostly good glycaemic control at the time of data collection. The mean levels of HbA1c were similar to other recent large cross-sectional observational studies of persons with T1DM in Spain [33,34]. However, a higher proportion of the study population presented with glycaemic control (HbA1c <7%, 53%) compared with those studies (22%-30%).

The finding that TIR was associated with some aspects of diabetes-related QoL (DQOL), but not with general QoL (EQ-5D-5L), is consistent with a previous study, which found that CGM contributes to significant improvement in diabetes-specific QoL, such as diabetes distress or fear of hypoglycaemia, in adults with T1DM, but not with QoL measures not specific to diabetes [35]. Also, a recent review showed that diabetes- and hypoglycaemia-specific, but not generic, PROs show the benefits of CGM and insulin pump technologies in persons with T1DM [36]. In our study, a weak but significant correlation was found between TIR and the diabetes-related concern subscale of DQOL (p=0.013). Prior studies have shown that fear of hypoglycaemia could be a major limitation for QoL and an active lifestyle in persons with T1DM [37-39]. However, in contrast with previous data, recent studies have shown that children and adolescents with fear of hypoglycaemia had a more active lifestyle and similar glycaemic metrics (including TIR) compared with those without fear [40-42]. Also, in a study among young Germans, CGM use was not associated with reduced diabetes distress or better glycaemic control [25].

Although the total cost derived from the use of healthcare resources during the previous six months was not significantly associated with TIR, the number of emergency room visits was negatively correlated with higher TIR. However, given the very low number of emergency room visits, this result should be taken with caution. The uptake of CGM by persons with T1DM has been associated with lower rates of emergency room visits or hospitalisations for hypoglycaemia in previous large cohort studies [43].

A recent study has suggested that CGM data sharing improves the QoL of both persons with T1DM and caregivers [44,45]. These benefits depended on the collaborative actions taken by persons with T1DM and caregivers to support diabetes management. Therefore, the use and understanding of CGM data in the form of easy-to-understand GPs such as TIR, TAR, or TBR, should be encouraged among persons with T1DM, and include caregivers and healthcare personnel. PROs should become valuable tools to evaluate progress towards glycaemic targets and to inform shared decision-making in clinical settings. However, it has been suggested that to better understand the association between GPs and QoL it is necessary to focus on the precise timing between the assessments of glucose and PROs, as ideally they should be assessed at the same time when they actually occur [26]. Also, in adults with T1DM, some studies have identified gaps in training and potential avenues for enhancing device education and CGM onboarding support [46]. Education after the uptake of CGM technologies could potentially help in reducing anxiety and improving QoL in persons with T1DM using CGM, but further studies are needed [47,48].

The study presents some limitations inherent to its design and the characteristics of the population included in it. As some variables were collected retrospectively, findings were limited to those data available in clinical practice electronic medical records, and information may be incomplete or inaccurate; however, they were data collected during routine practice and necessary for patient management. Further, the sample may not be representative of the population with T1DM in Spain as it comes from only four hospitals with specialised diabetes units, and PRO variables were collected in those patients who agreed to participate, who could have different characteristics from patients not willing to participate (e.g. the level of treatment satisfaction); however, as mentioned before, the mean glycaemic control was similar to other recent large cross-sectional observational studies of persons with T1DM in Spain. Since reported results regarding QoL were derived from self-reported data, they are based solely on participants’ memories and open to recall bias. Additionally, different time periods were considered for the various parameters of the study (14 days for CGM, six months for HCRU, or cross-sectional for PROs), which could limit the interpretation of the results. Although the PRO measures have limitations, the investigation of the association of these measures with GPs is of high importance in the current management of chronic conditions (patient-centred care).

## Conclusions

To the best of our knowledge, this is the first study that has systematically explored the association between TIR and PROs related to QoL (general and diabetes-related). The patients with T1DM participating in the study had mostly good glycaemic control. It was observed that good glycaemic control as measured by high TIR and low TAR1 is favourably associated with some aspects related to QoL when a specific questionnaire for diabetes was used (i.e. DQOL). Also, an association between higher TIR and with lower number of emergency room visits was observed.
